# Multi-joint gait clustering for children and youth with diplegic cerebral palsy

**DOI:** 10.1371/journal.pone.0205174

**Published:** 2018-10-24

**Authors:** Gregor Kuntze, Alberto Nettel-Aguirre, Gina Ursulak, Ion Robu, Nicole Bowal, Simon Goldstein, Carolyn A. Emery

**Affiliations:** 1 Faculty of Kinesiology, University of Calgary, Calgary, Alberta, Canada; 2 Department of Pediatrics, Cumming School of Medicine, University of Calgary, Calgary, Alberta, Canada; 3 C.H. Riddell Movement Assessment Center, Alberta Children’s Hospital, Calgary, Alberta, Canada; 4 Schulich School of Engineering, University of Calgary, Calgary, Alberta, Canada; 5 Section of Pediatric Orthopaedic Surgery, Alberta Children’s Hospital, Calgary, Alberta, Canada; Toronto Rehabilitation Institute - UHN, CANADA

## Abstract

**Background:**

Clinical management of children and youth with cerebral palsy (CP) is increasingly supported by computerized gait analysis. Methods have been developed to reduce the complexity of interpreting biomechanical data and quantify meaningful movement patterns. However, few methods are inclusive of multiple joints and planes of motion, and consider the entire duration of gait phases; potentially limiting insight into this heterogeneous pathology. The objective of this study was to assess the implementation of k-means clustering to determine clusters of participants with CP based on multi-joint gait kinematics.

**Methods:**

Barefoot walking kinematics were analyzed for a historical cohort (2007–2015) of 37 male and female children and youth with spastic diplegic CP [male n = 21; female n = 16; median age = 12 (range 5–25) years; Gross Motor Function Classification System Level I n = 17 and Level II n = 20]. Mean stance phase hip (sagittal, coronal, transverse), knee (sagittal), and ankle (sagittal) kinematics were time (101 data points), mean and range normalized. Normalized kinematics data vectors (505 data points) for all participants were then combined in a single data matrix M (37x505 data points). K-means clustering was conducted 10 times for all data in M (2–5 seeds, 50 repetitions). Cluster quality was assessed using the mean Silhouette value ($\bar{s}$) and cluster repeatability. The mean kinematic patterns of each cluster were explored with respect to a dataset of normally developing (ND) children using Statistical Parametric Mapping (SPM, alpha 0.05). Differences in potentially confounding variables (age, height, weight, walking speed) between clusters (C) were assessed individually in SPSS (IBM, USA) using Kruskal-Wallis H tests (alpha 0.05).

**Results:**

Four clusters (n_1_ = 5, n_2_ = 12, n_3_ = 12, n_4_ = 8) provided the largest possible data separation based on high cluster repeatability (96.8% across 10 repetitions) and comparatively greater cluster quality [$\bar{s}$ (SD), 0.275 (0.152)]. Participant data with low cluster quality values displayed a tendency toward lower cluster allocation repeatability. Distinct kinematic differences between clusters and ND data were observable. Specifically, C1 displayed a unique continuous hip abduction and external rotation pattern. In contrast, participants in C2 moved from hip adduction (loading response) to abduction (mid to terminal stance) and featured a unique ankle plantarflexor pattern during pre-swing. C3 was characterized by gait deviations in the sagittal plane of the hip, knee and ankle only. C4 displayed evidence for the most substantial hip and knee extension, and ankle plantarflexion deficit from midstance to pre-swing.

**Discussion:**

K-means clustering enabled the determination of up to four kinematic clusters of individuals with spastic diplegic CP using multi-joint angles without a priori data reduction. A cluster boundary effect was demonstrated by the Silhouette value, where data with values approaching zero were more likely to change cluster allocation. Exploratory analyses using SPM revealed significant differences across joints and between clusters indicating the formation of clinically meaningful clusters. Further work is needed to determine the effects of including further topographical classifications of CP, additional biomechanical data, and the sensitivity to clinical interventions to assess the potential for informing clinical decision-making.

## Background

Cerebral palsy (CP) describes a group of permanent disorders of the development of movement and posture, causing activity limitations, that are attributed to non-progressive disturbances that occurred in the developing fetal or infant brain [1]. Estimates of the world-wide prevalence of CP range from 1.5 to 4 per 1,000 new born children [2]. Children and youth with CP identify safe and efficient mobility as a primary concern to enable participation in activities of daily living [3]. Clinically, the examination of the structural aspects of gait for individuals with CP is increasingly aided by the use of motion analysis to accurately quantify the six-degree-of-freedom gait kinematics (i.e. segment and joint movement patterns) and kinetics (i.e. joint loading patterns). When utilized within the framework of an integrated clinical assessment [4], this approach has been shown to inform clinical decision-making [5,6] and support patient care [7].

Strategies to identify differences in gait kinematics and kinetics include: the description of segment and joint-specific deviations away from corresponding patterns for typically developing children [8]; the use of gait deviation indexes that provide continuous scores of movement deviations [9]; and the classification of gait patterns to form groups of individuals based on common movement characteristics [10]. However, a complete description of segment and joint-specific deviations away from a reference population may be challenging in light of the great number of clinically relevant gait patterns. Specifically, Nieuwenhuys et al. [8] identified 49 clinically important gait patterns across the pelvis, hip, knee and ankle in a recent Delphi consensus study. Importantly, rater experience was a concern as less experienced raters may be less reliable at identifying patterns at both the knee and ankle [11], contributing to the potential for different intervention recommendations [12].

Indexes of gait quality, such as the Gait Deviation Index [9], reduce data complexities by extracting discrete variables at select time points in the gait cycle to provide scores of overall movement deviation [13]. Indexes provide crucial information of gait quality and are sensitive outcome measures to investigate changes due to interventions [14]. However, as a single score they generally lack information on the segment-specific contributions to overall gait deviations. An exception is the Gait Profile Score [15], which retains information on segment and joint-specific deviations. However, the interpretation of the impact of such deviations on a patient-specific basis is inherently subjective.

Gait classifications of qualitative and quantitative data are used to group individuals into broader categories based on pre-defined criteria [16]. However, classification approaches are often based on partial information (e.g. sagittal plane only) or fail to provide sufficient information regarding their validity and reliability [16]. Further, classification based on the position of a single value to represent an individual may become problematic as the data variance within the individual is constrained. Therefore, data close to the boundary between classes could be erroneously classified. Alternative approaches are therefore needed to reduce the analysis burden, minimize rater bias, and reliably determine gait pattern deviations using clinically relevant gait data.

Machine learning provides a means to identify patterns in complex and multivariate data, while limiting rater bias. Both supervised [17] and unsupervised approaches [18] have been implemented to objectively delineate differences in gait for individuals with CP. Supervised machine learning is based on the training of a classifier using input data for known groups. This classifier may then be used to enable the classification of new data of unknown group allocation to one of typically two clusters (i.e. binary classification). In contrast, unsupervised learning does not require a priory knowledge of group allocation but enables the determination of the number of clusters contained within a dataset. This may be regarded as beneficial for the purpose of identifying how many clusters are present in a diverse population of children with CP and which data features allowed for cluster differentiation. Therefore, machine learning approaches have the capacity to retain the underlying information that enabled data classification and provide a means to assess the quality of data classification [19,20]. Yet, concerns regarding gait classification persist with respect to content validity (i.e. inclusion of limited gait information; limited sample size), construct validity (i.e. specific rationale for chosen number of clusters), and lack of information regarding reliability [16]. Consequently, there is a continuing need for the evaluation of machine learning approaches for gait biomechanics in clinical populations. Additional information is needed regarding the quality of gait clusters for individuals with CP, and the characteristics of individuals within and differences between clusters, to better understand the potential for the clinical utility of machine learning approaches.

The objective of this study was to assess the implementation of an unsupervised machine learning approach (k-means clustering) to determine clusters of children and youth with spastic diplegic CP based on lower limb gait kinematics. Specifically, this study investigated how many clusters were observed, using measures of cluster quality and repeatability, and which corresponding kinematics features contributed to the separation of gait patterns for individuals in this cohort. Further, the kinematic characteristics of individuals allocated to the observed clusters were compared to those of normally developing children to describe the corresponding gait deviations.

## Methods

### Participants

Data analysis was conducted for a historical cohort of male and female children and youth with a diagnosis of diplegic CP [n = 37; male n = 21; female n = 16; median age = 12 (range 5–25) yrs; Gross Motor Function Classification System Level I n = 17 and Level II n = 20]. All participants performed a gait assessment as part of a pre-surgical clinical consultation for complex intervention planning between 2007 and 2015. This study was a secondary analysis that did not require child or parent consent. Ethics approval for this study was received by the Conjoint Health Research Ethics Board of the University of Calgary (REB15-2825).

### Gait analysis

Participants conducted a series of barefoot walking trials at a self-selected speed and without the use of assistive devices along the length of a raised wooden walkway (9.9 x 1.8 m, expanded to 9.9 x 4.2 m in 2010) under the supervision of a physiotherapist. First, reflective spherical markers were affixed to the lower extremities and trunk of the participants using a modified Helen-Hayes marker set. Gait kinematics were then recorded using an 8-camera optical motion analysis system (Motion Analysis, USA) that was expanded to 12-cameras in 2010. Ground reaction forces were collected synchronously (1200 Hz) with gait kinematics (120 Hz) using two OR6-6 force plates (AMTI, USA), expanded to four OR6-6 force plates in 2010, embedded in the walkway. Five successful gait repetitions were identified for each participant. Successful gait repetitions consisted of a completely visible marker set, contact of one foot only with the center of one of the force plates, and maintenance of expected gait dynamics, verified using visual inspection. Marker data were tracked using EVART (Motion Analysis, USA). Stance phase hip, knee and ankle joint angles were computed using Visual-3D (C-Motion, USA) and mean walking speeds were determined in Visual-3D as the mean stride length over stride time across five gait repetitions.

### Data clustering

#### Data matrix

**Fig 1:**
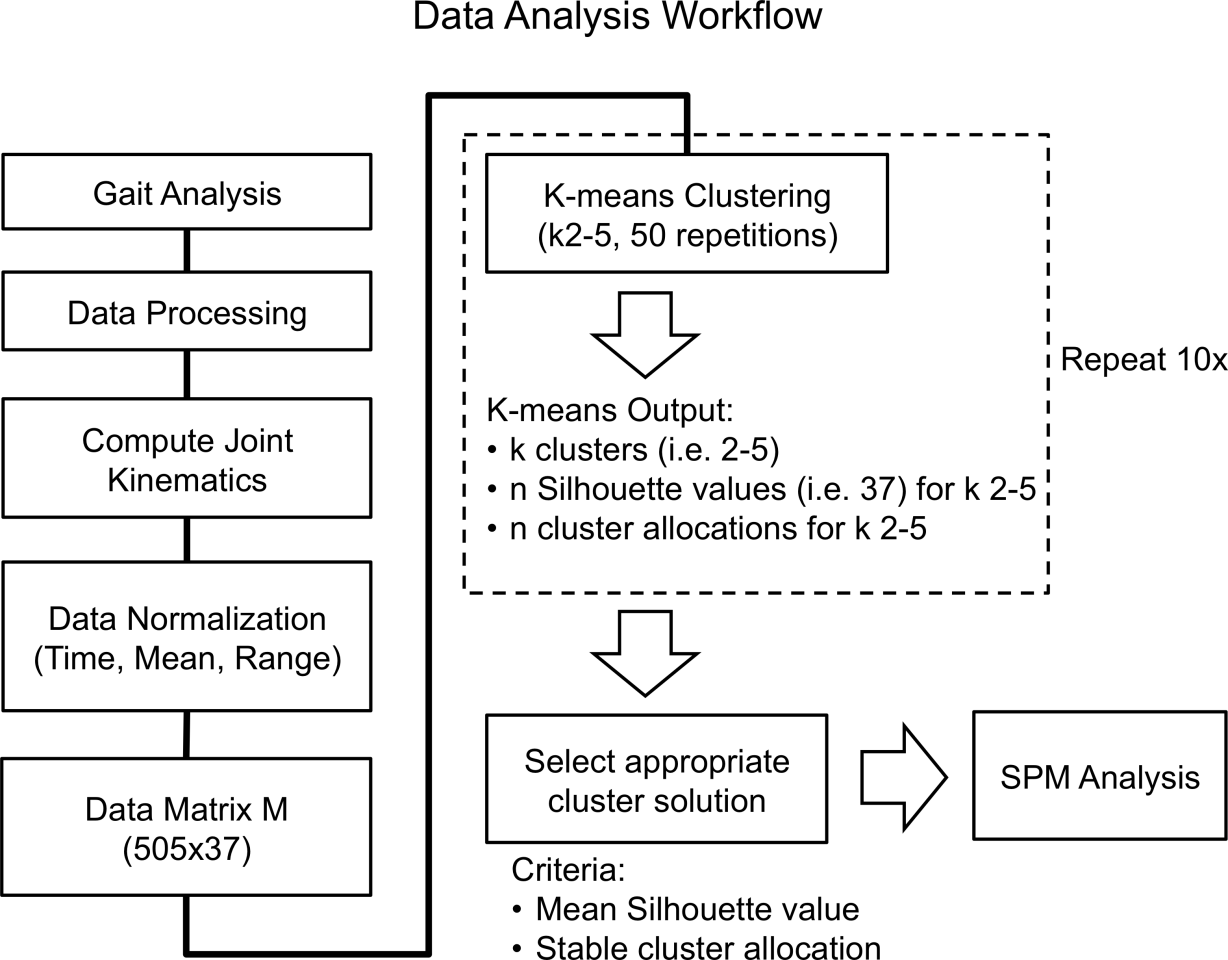
Illustration of the data analysis workflow employed in this study.

Hip (sagittal, coronal, transverse), knee (sagittal), and ankle (sagittal) joint angle time series (i.e. stance phase) of the left leg for each participant were taken forward for analysis (Fig 1). The selection of joints and planes of motion was based on an international expert consensus of clinically important joint patterns for children with CP, developed using Delphi surveys [8]. All kinematics data were time normalized to 101 data points from heel strike (HS) to toe-off (TO). HS and TO events were automatically identified in Visual-3D using ground reaction force data. The mean kinematic patterns at each joint and plane of motion were computed across the five selected steps for each participant. Prior to data clustering, all participant-specific mean kinematic patterns were mean and range normalized by subtracting the respective mean values of the kinematic patterns and dividing by their standard deviations. Thereafter, an input data vector was created for each participant consisting of the 101 normalized data points for each joint and plane of motion. Therefore, each participant was represented by a data vector D (i.e. 505x1 data points) and all participant data vectors were combined in a data matrix M (i.e. 505x37). The order of joints and planes of motion was consistent across all participant data in data matrix M.

#### Mean pattern clustering

Data clustering was performed in Matlab (v.2016b, MathWorks, USA) using the k-means clustering algorithm by Alhoniemi (http://www.cis.hut.fi/somtoolbox/). K-means clustering is an iterative process whereby the distances between input data and a number of randomly allocated seeds (k) in the data space are determined. This results in k temporary clusters of data allocated to each seed. Next, the mean of all data contained within each temporary cluster (i.e. the cluster centroid) is calculated and, in turn, is allocated as a new seed and again clusters are formed based on the distance of the data to the seeds. This process of re-defining the cluster centroids is repeated until the change in position of the cluster centroids converges on a constant location. Due to the random initial allocation of seeds, the k-means algorithm may not reach the ideal global optimum at any one iteration but may instead converge on an undesirable local optimum. Therefore, the clustering solution may be dependent on the number of seeds and the initial starting point of each randomly allocated seed. To overcome this limitation, multiple iterations of k-means are run with altering seed locations and an optimal solution is chosen based on the sum of squared errors as a selection criterion. Here, the error is the Euclidian distance of each data point to the nearest cluster centroid and the smallest squared error is regarded as an optimal clustering solution. Within this implementation of k-means clustering, 2–5 seeds (k) were chosen, 50 repetitions were conducted for each fixed number of initial seeds, and the Euclidean distance of data to cluster centroids was used as the distance function. Further, this clustering process was repeated 10 times to investigate differences in the cluster allocations of individual data vectors for increasing seed numbers over consecutive iterations of k-means clustering.

#### Cluster selection

Cluster quality was assessed using the Silhouette function by Rousseeuw [20] in Matlab. The Silhouette determines the relative distance of each data vector with respect to its own and all other clusters. Specifically, the Silhouette value (*s*) provides a measure of the similarity of data within a cluster and with respect to all other clusters using a scale of -1 to 1:

$$s(i)=\frac{b(i)-a(i)}{max\{a(i),b(i)\}}$$ (Eq 1)

where, s(i) is the Silhouette value; a(i) is the mean distance of participant datum with respect to all other data in its cluster; and b(i) is the lowest mean distance of i to any other remaining cluster. Therefore, greater positive values indicate a stronger association of data to their allocated cluster (cohesion) and negative values indicate a greater similarity to other clusters. In this implementation, the squared Euclidean distance was chosen for calculating a(i) and b(i) and Silhouettes were visualized for k 2–5 (Fig 1). Cluster quality was assessed by computing the mean value of *s* (${\bar{s}}_{k}$) for all data in k 2–5 and comparing the differences in ${\bar{s}}_{k}$ across increasing cluster numbers. In addition, clustering repeatability was assessed by tracking the change in data allocations with increasing k. Specifically, cluster allocations and their respective *s* values were recorded for each input data vector across the 10 k-means clustering repetitions. Finally, the selection of an optimal cluster solution was based on the number of clusters that separated the data into the largest number of groupings while retaining high cluster quality and repeatability (i.e. percentage of data not changing cluster allocation).

#### Cluster assessment

Following selection of a cluster solution that separated data into the highest number of clusters, the kinematic characteristics of the hip, knee, and ankle for each cluster were identified with respect to dataset of normally developing (ND) children. ND data consisted of four unique mean kinematic waveforms for the hip, knee and ankle for individuals aged 5 to 11 years (Motion Analysis, USA). In order to provide representative data variances for each normative dataset, four additional waveforms were generated at ±0.5 and ±1 times the standard deviation (SD) for each joint. This resulted in a total of 20 waveforms (i.e. 4 means x 5 SDs) for the hip (sagittal, coronal, transverse), knee (sagittal) and ankle (sagittal) for comparison with corresponding cluster-specific joint patterns using Statistical Parametric Mapping (SPM, www.spm1d.org) implemented in Matlab. SPM is a statistical approach to conduct hypothesis testing across the time domain [21]. The SPM approach can be viewed as consisting of two steps: (1) the computation of a scalar output statistic at each point in the data series (SPM{t}); and (2) the definition of a critical threshold. SPM{t} indicates the magnitude of the difference between two sets of waveforms. The critical threshold is derived by considering all points or scalars from all locations and provides a measure of the probability that differences between the sets of waveforms are larger than those expected from repeated random sampling. Therefore, any area of SPM{t} that exceeds the critical threshold, determined using random field theory and specified alpha, indicates a significant difference between sets of waveforms. Additionally, analyses of the difference in potentially confounding variables between kinematic clusters were conducted. Variables of interest included: age, height, weight, and walking speed.

### Statistics

Statistical analyses were conducted using Matlab and SPSS (IBM, USA). Kinematic deviations between participant data allocated to each cluster and a dataset of ND children were assessed descriptively with the aid of SPM in Matlab as outlined above (alpha 0.05). Further, analyses of differences in potentially confounding variables between clusters were conducted using Kruskal-Wallis H tests in SPSS (alpha 0.05).

## Results

### K-means clustering

**Fig 2:**
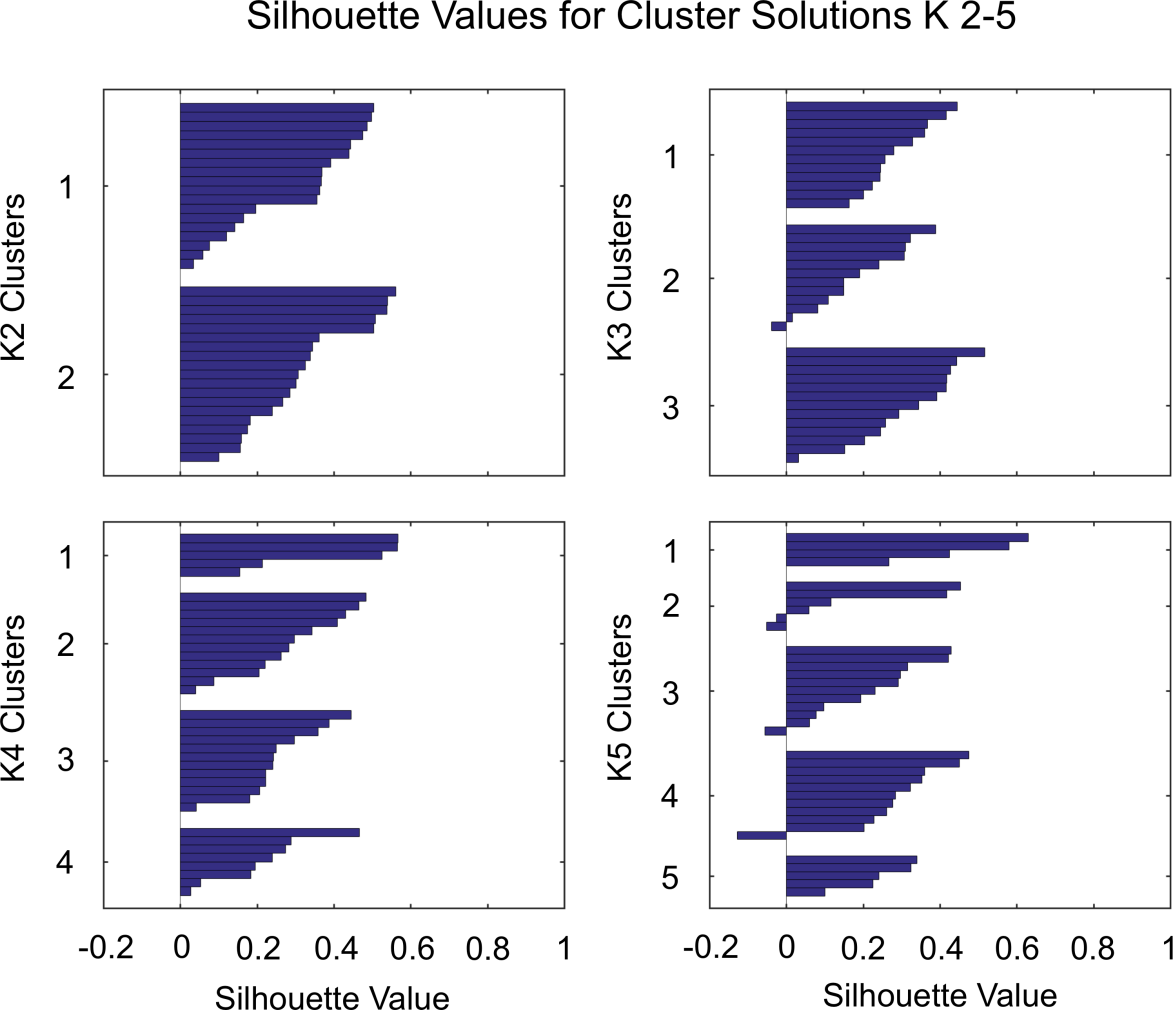
Summary of Silhouette values for k 2–5. K2 and K3 resulted in identical data allocations for every repetition of k-means clustering. Due to a more variable cluster allocation for K4 the median cluster allocation is presented here. K5 cluster allocations displayed poor repeatability and a representative clustering outcome is presented.

**Fig 3:**
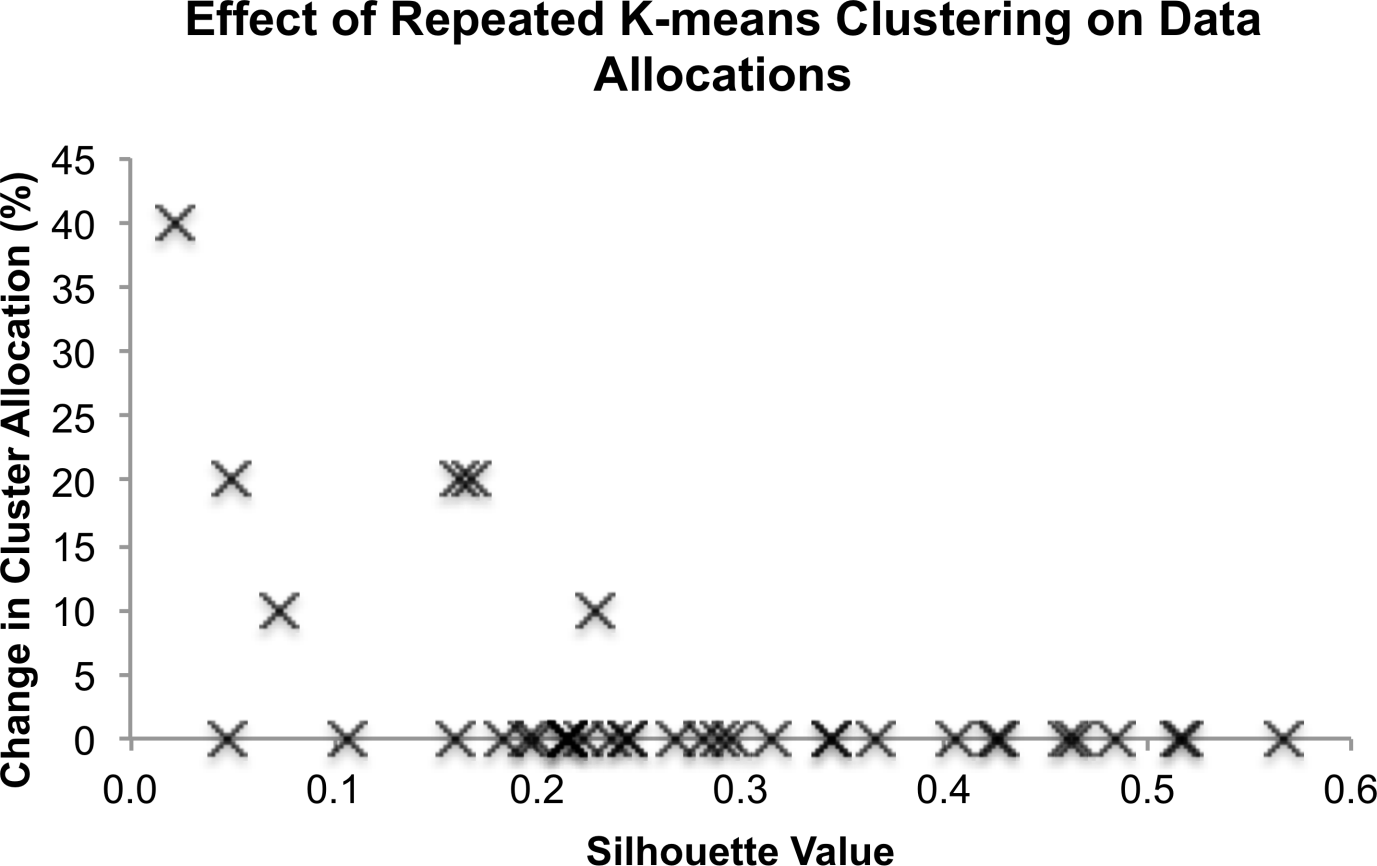
The effect of repeating k-means clustering ten times is presented with respect to the change in cluster allocation and the respective mean Silhouette value. Across repetitions, data for two individuals changed cluster allocations once, three individuals changed twice, and one individual changed four times. As expected, the probability for individuals to change cluster allocations increases with decreased mean Silhouette values.

K-means clustering results for k 2–5 are summarized in Fig 2. Mean Silhouette values ($\bar{s}$) across ten k-means cluster repetitions were [mean (SD)]: k = 2 [0.316 (0.154)]; k = 3 [0.268 (0.132)]; k = 4 [0.275 (0.152)]; and k = 5 [0.256 (0.173)]. Further analysis of cluster allocations of individual data vectors across 10 consecutive clustering repetitions indicated that all participant data were allocated to the same clusters for k = 2 and k = 3. Data allocations to individual clusters were more variable for k = 4 with 6 out of 37 individuals switching between clusters at least once (Fig 3). Across 10 cluster repetitions a total of 12 out of 370 cluster allocations (i.e. 3.2%) were observed to switch between clusters. The likelihood of data being allocated to different clusters increased with decreasing values of *s*, in line with data being located close to the cluster boundary (Fig 3). No consistent pattern of cluster allocations could be identified for the k = 5 solution. These findings indicate that the two- and three-cluster solutions may be comparatively conservative, while the five-cluster solution does not provide repeatable data allocations. The four-cluster solution may be regarded as an optimal clustering solution for separating the current dataset into the largest number of clusters given the comparatively greater cluster quality and high repeatability (96.8% repeatability). Consequently, the four-cluster solution was taken forward for further analysis to examine the characteristics of joint kinematic patterns of individuals allocated to each cluster. Due to the switching of some participants between clusters, it was necessary to determine the most likely cluster allocation for these individuals. Consequently, the median cluster allocation for each participant across the 10 k-means repetitions was computed for the four-cluster solution and the resultant cluster IDs were then used for the remainder of the data analysis.

### Cluster characteristics

**Fig 4:**
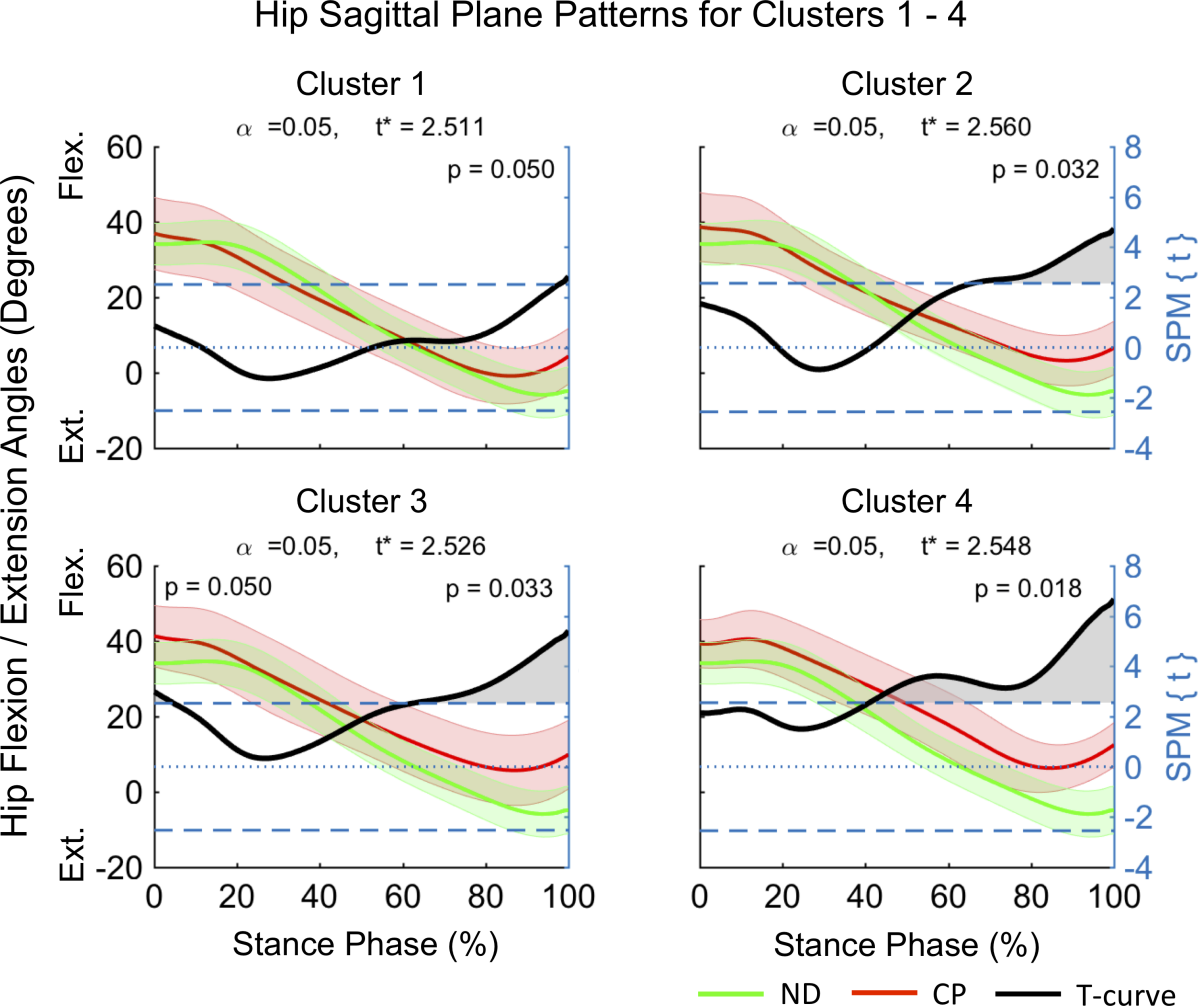
Sagittal plane kinematics of the hip for participants allocated to clusters 1–4. Mean data for participants with CP (red line) and normally developing children (green line) are shown (left vertical axis) for the duration of stance phase. Results from SPM analysis (black line) are shown with respect to the right axis. Shaded gray areas indicate significant differences between clusters and ND data. Abbreviations: Flexion (Flex), Extension (Ext).

**Fig 5:**
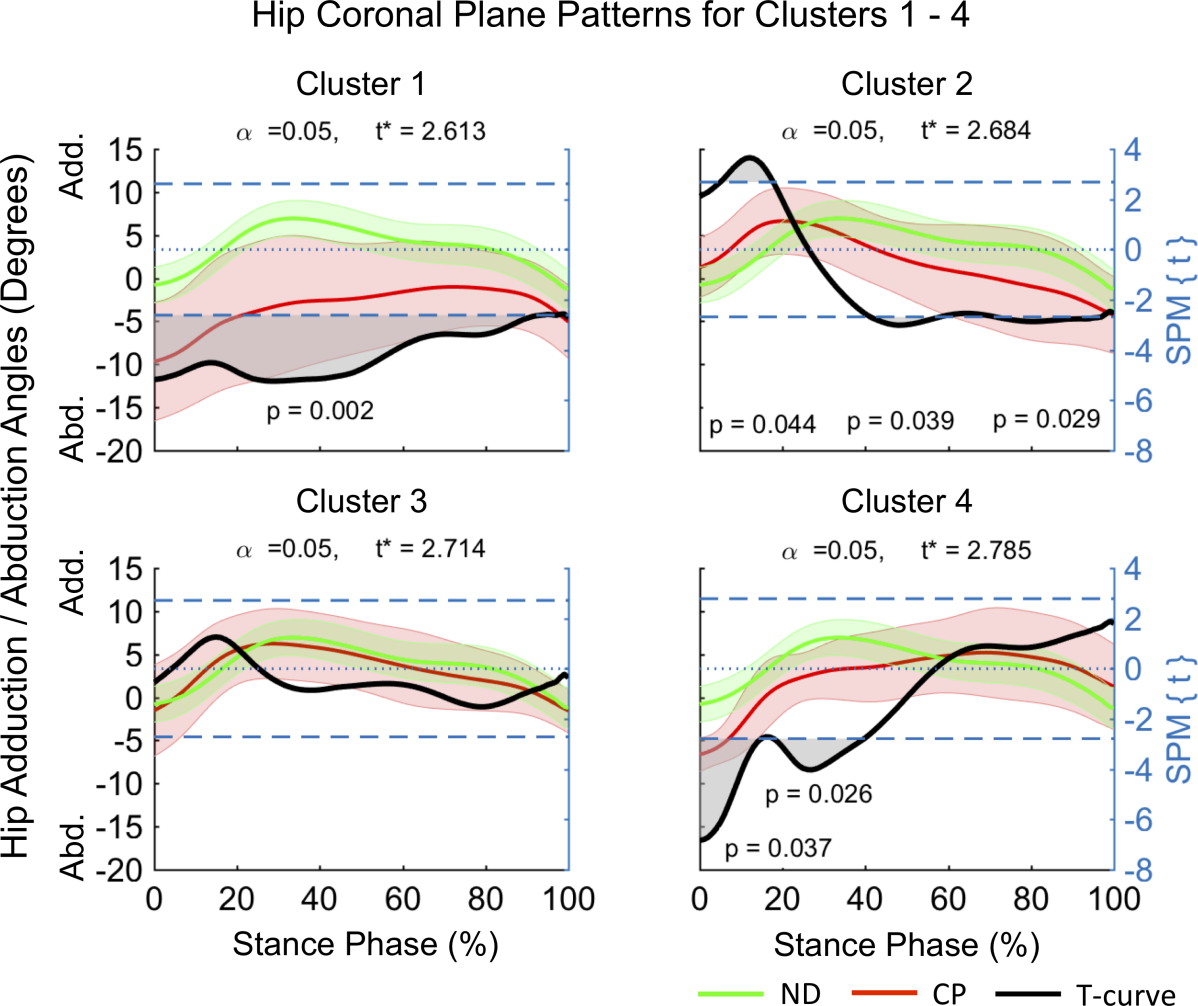
Coronal plane kinematics of the hip for participants allocated to clusters 1–4. Mean data for participants with CP (red line) and normally developing children (green line) are shown (left vertical axis) for the duration of stance phase. Results from SPM analysis (black line) are shown with respect to the right axis. Shaded gray areas indicate significant differences between clusters and ND data. Abbreviations: Adduction (Add), Abduction (Abd).

**Fig 6:**
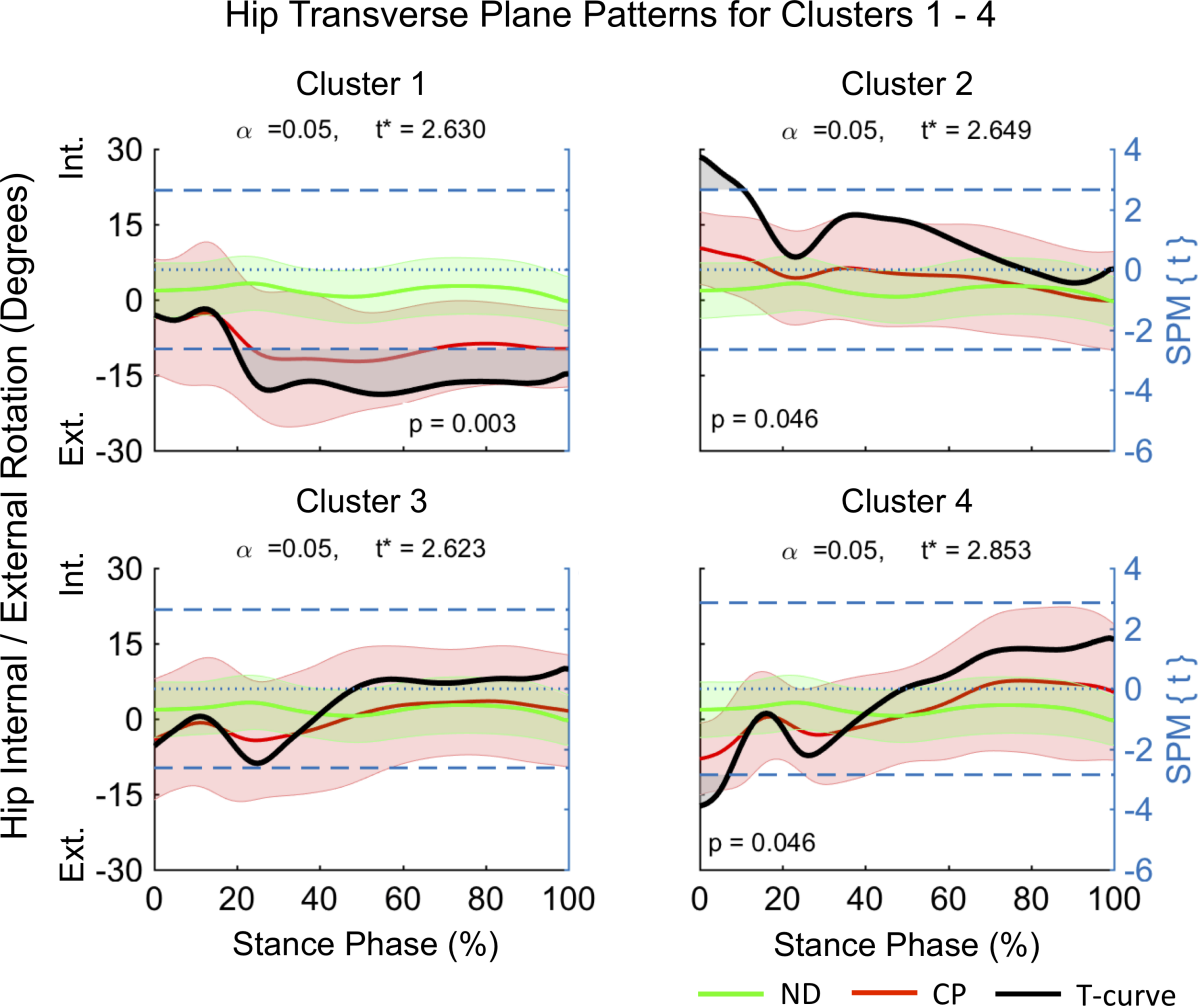
Transverse plane kinematics of the hip for participants allocated to clusters 1–4. Mean data for participants with CP (red line) and normally developing children (green line) are shown (left vertical axis) for the duration of stance phase. Results from SPM analysis (black line) are shown with respect to the right axis. Shaded gray areas indicate significant differences between clusters and ND data. Abbreviations: Internal Rotation (Int), External Rotation (Ext).

Clusters (C) 1–4 consisted of n_1_ = 5, n_2_ = 12, n_3_ = 12 and n_4_ = 8 participants. SPM analysis outcomes of the differences between mean joint kinematic patterns for each cluster and a ND dataset are summarized in Fig 4–Fig 8 and Table 1. At the sagittal plane of the hip all clusters indicated an extension deficit compared with ND patterns (Fig 4). The duration of this deficit differed between clusters (Table 1) indicating an increase in deficit magnitude for C1 (98–100% stance), C2 (66–100% stance), and C4 (41–100% stance). C3 was the only cluster indicating a hip extension deficit from HS until early loading response (0–5% stance) and from terminal stance until TO (63–100% stance). At the coronal plane the hip was in greater abduction for C1 and C4 (Fig 5). However, while C1 displayed evidence for continuous hip abduction for the majority of stance (0–94% stance), differences for C4 were no longer evident after midstance (0–40% stance, Table 1). C2 was characterized by a change from greater hip adduction during loading response (5–18% stance) to greater hip abduction at mid to terminal stance (41–60% stance). No pattern differences were observed for C3. Pattern differences in the transverse plane of the hip (Fig 6) were most pronounced for C1, which displayed a continuous external hip rotation (20–100% stance). In contrast, C2 and C4 displayed evidence for greater early internal (0–11% stance) and external (0–7% stance) hip rotation, with no pattern differences observed for C3.

**Table 1:** Summary of mean kinematic pattern deviations for data allocated to each cluster with respect to a dataset of normally developing children. The direction of observable pattern deviations and the timing of the deviations are indicated with respect to percent stance phase of walking.

	Hip						Knee		Ankle	
	Sagittal		Coronal		Transverse		Sagittal		Sagittal	
Cluster	Deviation	% Stance	Deviation	% Stance	Deviation	% Stance	Deviation	% Stance	Deviation	% Stance
**1** (n = 5)	↑ Flex.	98–100	↑ Abd.	0–94	↑ Ext. Rot.	20–100	↑ Flex.	0–18	↑ Dorsi.	8–44
**2** (n = 12)	↑ Flex.	66–100	↑ Add.	5–18	↑ Int. Rot.	0–11	↑ Flex.	0–20	↑ Dorsi.	8–31
			↑ Abd.	41–60					↑ Plant.	90–100
**3** (n = 12)	↑ Flex.	0–5	N/A		N/A		↑ Flex.	0–18	↑ Dorsi.	10–28
		63–100						96–100		
**4** (n = 8)	↑ Flex.	41–100	↑ Abd.	0–15	↑ Ext. Rot.	0–7	↑ Flex.	0–18	↑ Dorsi.	11–33
				18–40				60–100		49–86

**Fig 7:**
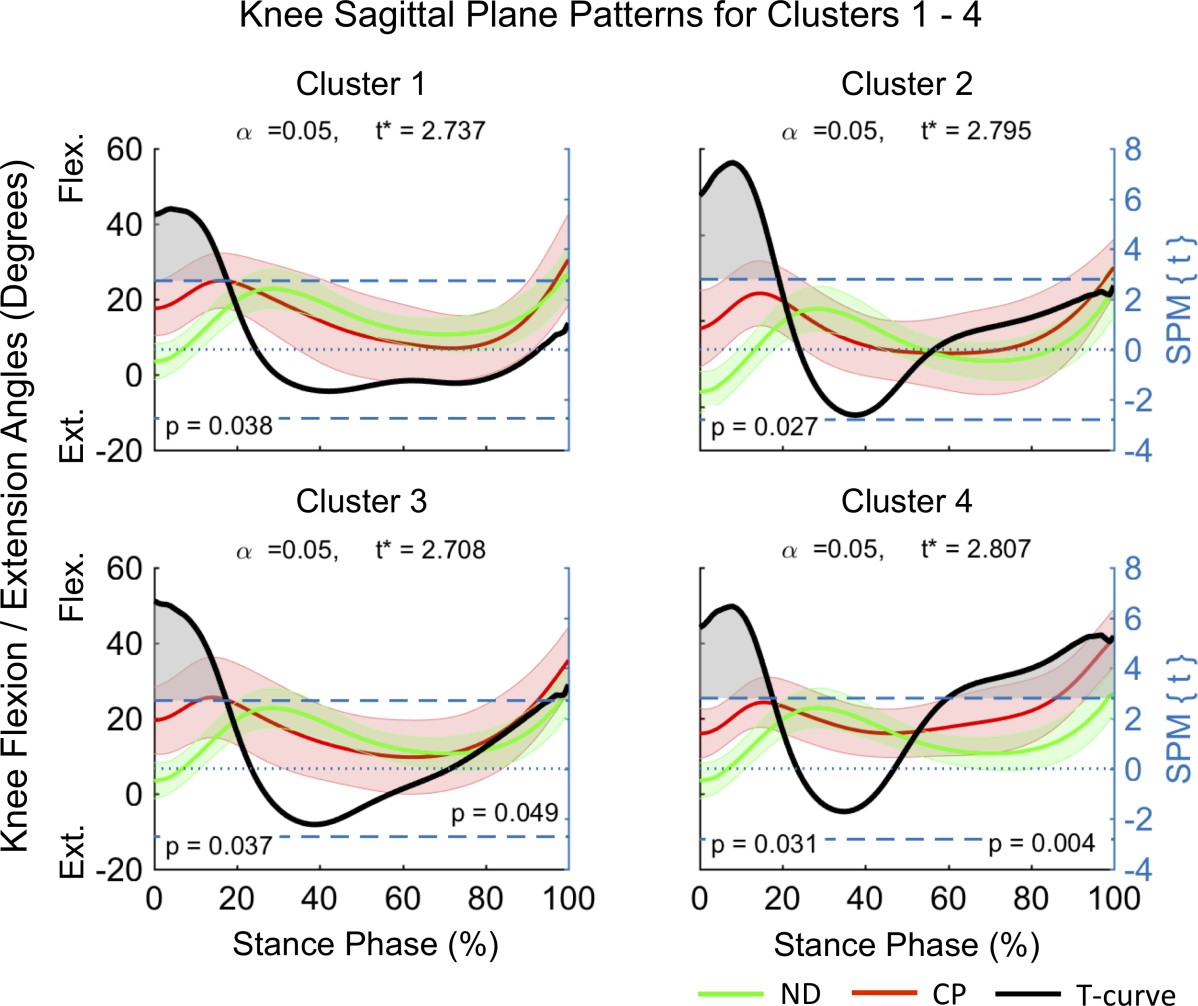
Knee sagittal plane kinematics for participants allocated to clusters 1–4. Mean data for participants with CP (red line) and normally developing children (green line) are shown (left vertical axis) for the duration of stance phase. Results from SPM analysis (black line) are shown with respect to the right axis. Shaded gray areas indicate significant differences between clusters and ND data. Abbreviations: Flexion (Flex), Extension (Ext).

**Fig 8:**
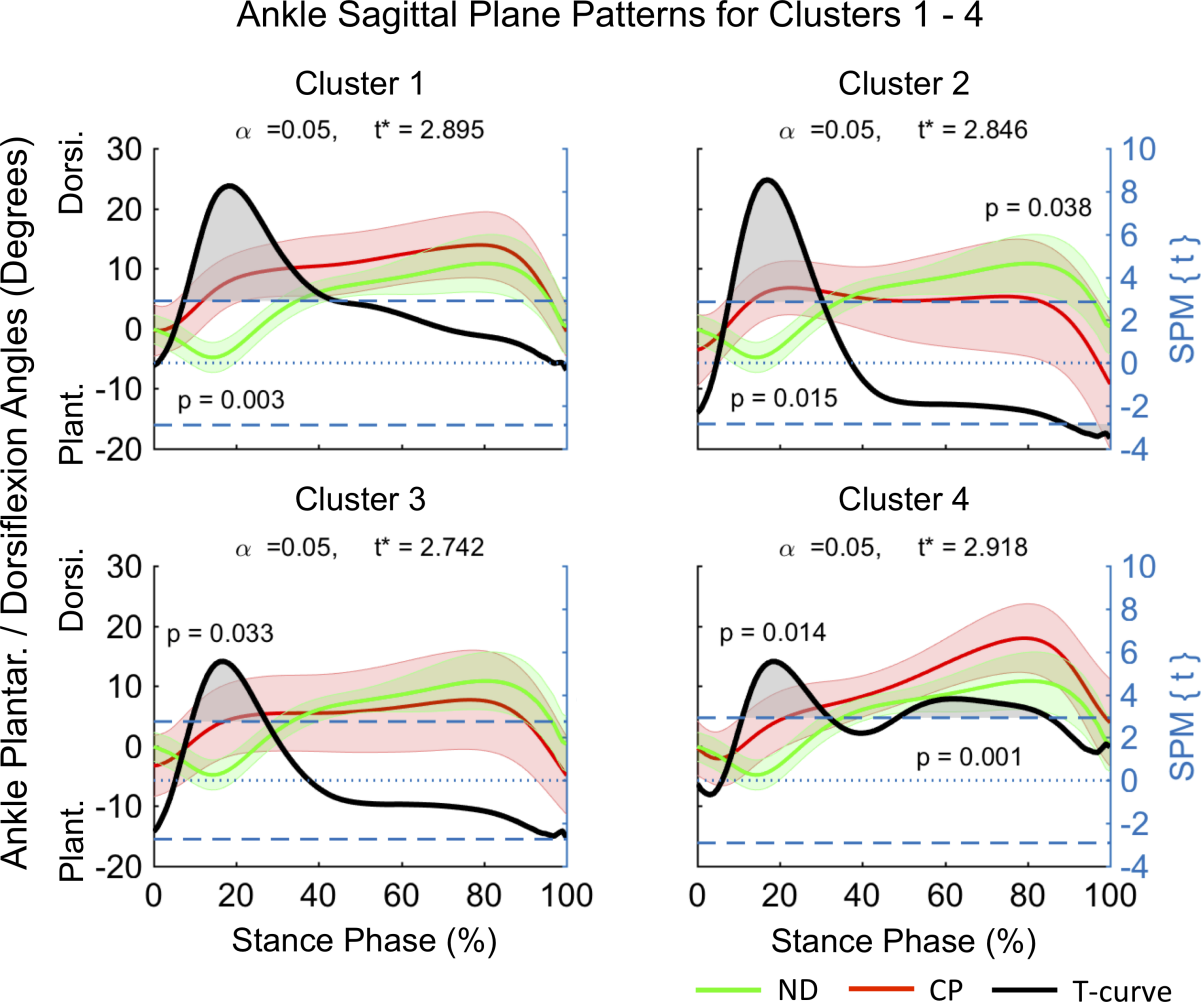
Ankle sagittal plane kinematics for participants allocated to clusters 1-4. Mean data for participants with CP (red line) and normally developing children (green line) are shown (left vertical axis) for the duration of stance phase. Results from SPM analysis (black line) are shown with respect to the right axis. Shaded gray areas indicate significant differences between clusters and ND data. Abbreviations: Dorsiflexion (Dorsi), Plantarflexion (Plant).

Pattern differences at the sagittal knee indicated a knee extension deficit for all clusters (Fig 7). C1 and C2 displayed similar knee patterns with greater early knee flexion evident (0–18% and 0–20% stance respectively). In addition to an early knee extension deficit (0–18% stance), C3 and C4 displayed a further pattern of knee extension deficit at pre-swing (C3 96–100% stance) and from terminal stance to TO (C4 60–100%). At the sagittal ankle (Fig 8) all clusters displayed increased ankle dorsiflexion during loading response and midstance (8–44%, 8–31%, 10–28% and 11–33% stance for C1-4 respectively). An additional pattern of increased ankle dorsiflexion at mid to around pre-swing was observed for C4 (49–86% stance). In contrast, C2 provided evidence for increased ankle plantarflexion during pre-swing (90–100% stance).

### Confounding variables

Based on the outputs of the Kruskall-Wallis H tests, no differences in potentially confounding variables between clusters were identified: age (p = 0.324); height (p = 0.286); weight (p = 0.668); walking speed (p = 0.873) (Table 2).

**Table 2:** Summary of cluster characteristics [median and interquartile range (IQR)] with respect to the age, height, weight, and walking speed of individuals allocated to clusters 1–4.

	Age (yrs)	Height (m)	Weight (kg)	Walking Speed (m/s)
Cluster	Median (IQR)	Median (IQR)	Median (IQR)	Median (IQR)
**1**	12.00 (3.00)	1.57 (0.14)	42.0 (13.25)	1.05 (0.29)
**2**	9.00 (10.75)	1.43 (0.40)	35.80 (36.88)	0.97 (0.42)
**3**	7.00 (8.00)	1.35 (0.37)	41.00 (37.75)	1.06 (0.29)
**4**	13.00 (3.50)	1.55 (0.20)	44.25 (25.63)	0.93 (0.30)

## Discussion

The machine learning approach implemented in this study provided a means to successfully identify kinematic clusters of individuals with spastic diplegic CP. Specifically, measures of cluster quality and repeatability indicated that the selected dataset of lower limb gait kinematics could be separated into a maximum of four clusters. Resultant clusters displayed evidence for distinct and clinically meaningful joint angle pattern differences with respect to data for normally developing children. This methodology creates a platform to reveal additional knowledge on the consequences of brain disorders in children, quantify an individual’s response to changes in activity demands (e.g. fatigue, growth and development), and monitor the effects of physical therapy and surgical interventions.

A key challenge in the clinical interpretation of gait deviations in children and youth with CP is the management of large amounts of biomechanical data obtained from computerized motion analysis. Machine learning offers the potential to reduce the analysis burden and limit rater bias by using quantitative measures to group individuals with similar gait patterns. A key aspect of the chosen methodology was the inclusion of multi-joint data for the duration of stance phase without prior data reduction and the use of cluster repeatability, in addition to the Silhouette function, to identify the solution that separates the dataset into the largest number of distinct clusters. Data reduction techniques such as self-organizing maps have been shown to enhance cluster outcomes when implemented prior to data clustering [22]. However, information on any one individual is lost in the process as the reduced data are combinations of the individual patterns forming new variables. Basing the clustering approach on the full complement of clinically meaningful joint kinematics data therefore retained the ability to identify individuals with similarities in gait deviations, quantify the quality of their cluster allocation, and investigate the kinematics features that enabled the formation of the respective data clusters.

Four main gait clusters were identified based on excellent cluster repeatability (i.e. changes in cluster allocation) and comparatively higher cluster quality (i.e. mean Silhouette value, Fig 2). It can be argued that, despite higher repeatability values, the two- and three-cluster solutions provided rather conservative cluster estimates that failed to reveal all possible sub-groups within the dataset, while the five-cluster solution was rejected due to very poor repeatability. Specifically, the four-cluster solution resulted in slightly higher $\bar{s}$ values than the three-cluster solution, while only demonstrating a small reduction in cluster repeatability across individuals over 10 cluster repetitions (i.e. 3.2%). The slight reduction in repeatability was due to a switch in cluster allocations for six participants, who changed cluster allocations at least once (Fig 3). It is interesting to note that cluster switches were not random but that individuals only switched their allocation between two participant-specific clusters. These observations indicate that the separation of clusters reached a limit for the four-cluster solution where the probability of data falling on the margins between clusters increased. This effect is further illustrated by the increased likelihood of cluster switches for individuals with $\bar{s}$ values approaching zero, where $\bar{s}$ provides a numerical estimate of cluster margin proximity (Fig 3). Any additional clusters, on the other hand, did not allocate data in a repeatable manner and were thus rejected.

In order to investigate the kinematic characteristics of the four identified gait clusters a descriptive approach, supported by SPM analysis, was chosen. The largest clusters were C2 and C3, each containing ~32.4% of participants in this cohort, with C1 and C4 containing ~13.5% and ~21.6% of participants respectively. Distinct differences in the timing and duration of deviations in mean kinematic patterns of the hip, knee and ankle, with respect to ND data were observed within clusters (Fig 4–Fig 8, Table 1). C3 was characterized by gait deviations in the sagittal plane of the hip, knee and ankle only. The hip displayed some indication of greater initial flexion (0–5% stance) and a substantial extension deficit during terminal stance and pre-swing (63–100% stance). Similarly, the knee was in greater flexion during the loading response (0–18% stance) and again during pre-swing (96–100% stance), while the ankle was in greater dorsiflexion during loading response and midstance (10–28% stance). These pattern deviations are similar to those expected for an apparent equinus gait as defined by Rodda et al. [23], which is commonly associated with spasticity and contractures of the hamstrings and iliopsoas [24].

C2 displayed similar deviations at the sagittal hip (66–100% stance) and knee (0–20% stance), however, participants within this cluster further displayed evidence of a unique ankle plantarflexor pattern during pre-swing, indicative of a jump gait pattern [23]. Jump gait is the most common gait abnormality in children with spastic diplegia and is associated with increased hamstring and hip flexor spasticity [25] as well as spasticity of the triceps surae during weight acceptance [26]. In contrast to C3, additional adaptations at the coronal and transverse planes of the hip were observed indicating that participants initiated stance phase with greater hip adduction (5–18% stance) and internal rotation (0–11% stance) and then progressed to hip abduction in midstance. These findings may be a compensation to accommodate foot clearance during swing. Further, a resultant more medial foot placement during single limb support may be advantageous in order to accommodate potential gluteal muscle weakness. Assessment of the contributions of the trunk to the observed medial foot placement may reveal insights into whole body compensations.

C1 had the smallest hip extension deficit amongst clusters (98–100% stance), while displaying similar knee flexion (0–18% stance) and ankle dorsiflexion (8–44% stance) patterns as observed for C2 and C3. In contrast to the remaining clusters, C1 displayed evidence for the most pronounced deficits at the coronal (0–94% stance) and transverse (20–100% stance) planes of the hip (Fig 5 and Fig 6). Here, participants maintained an abducted and externally rotated hip for the majority of stance. These findings are in alignment with those by Nieuwenhuys et al. [27] who described a continuous hip abduction pattern and excessive hip external rotation pattern for 9.2% and 8.9% of a population of children with unilateral and bilateral spastic CP respectively. Anecdotally, participants in C1 displayed evidence for an internally rotated pelvis at the initiation of stance, as well as frontal plane motion at the knee and the ankle / foot complex. Further investigations are needed to better understand the causes and consequences of these gait abnormalities, such as the potential widening of the base of support during weight bearing.

C4 was unique amongst the four identified clusters, displaying the longest durations of hip (41–100% stance) and knee (0–18 and 60–100% stance) extension deficits, combined with a unique ankle dorsiflexion pattern at terminal stance and pre-swing (49–86% stance). Additionally, participants in C4 appeared to adopt similar hip strategies as those in C1, with significant hip abduction (0–15 and 18–40% stance) and hip external rotation (0–7% stance) deviations. This combination of substantial sagittal plane deviations across the hip, knee and ankle is in line with those of a crouch gait [23]. Specifically, the crouch gait pattern is associated with multifactorial contributions including ankle plantarflexor weakness, decreased selective motor control, increased spasticity, and flexion contractures at the knee and hip [25]. Indeed, hip flexor tightness may explain the observed coronal and transverse plane abnormalities, which may be due to a combination of a greater anterior pelvic tilt and internal pelvic rotation, placing the hip into greater abduction at initial contact to enhance stride length.

The similarities and distinct differences in kinematic patterns identified across clusters provide evidence for the ability of the chosen machine learning approach to extract clusters of individuals based on similarities in their gait patterns. Importantly, these data indicate that pattern deviations within clusters aligned well with respect to current consensus on typical gait patterns in spastic diplegic CP. Based on the data presented here, no differences in potential confounders (i.e. age, height, weight, walking speed) were observed. However, given the small sample size and existing evidence for the effects of growth and development on gait these variables cannot be rejected and additional research on the effects of growth and development in children with CP is needed.

## Limitations

The observations in this study are based on a small sample of pre-surgical individuals that does not reflect the full spectrum of gait deviations for individuals with CP or the consequences of surgical intervention. It is likely that the inclusion of further participants with different topographical classifications of CP and additional gait data will alter cluster characteristics. While efforts were made to include clinically meaningful joint patterns, information on pelvic orientation was not included and only the stance phase of the left limb was considered. Therefore no information regarding the complete gait cycle is currently available. Due to marker placement limitations, ankle joint kinematics in spastic CP have to be treated with caution as they may be confounded by foot deformities, calcaneal plantarflexion and midfoot break resulting in the appearance of a dorsiflexed ankle. Consequently, additional assessment may be required to clarify etiology and guide treatment plans. Further, it should be noted that individual joint patterns for any one participant may deviate from other cluster members. This is expected as this implementation of unsupervised clustering aimed to identify similar gait patterns across multi-joint data. Additional work is needed to explore the number and quality of kinematic clusters for a representative population of individuals with CP. Further, additional machine learning approaches, including supervised learning algorithm and neural networks, need to be explored to enable automated pattern recognition for implementation in the clinical domain.

## Conclusion

The findings of this study demonstrate that k-means clustering is a viable means to determine clusters of individuals with similar multi-joint gait patterns. Pattern deviations within clusters aligned with current opinions on clinically meaningful gait deviations. Further, by including coronal and transverse plane hip kinematics, it was possible to gain additional insights into gait strategies employed by participants in this cohort. These observations are encouraging for the development of automated solutions to inform surgical and non-surgical intervention planning. To work toward clinical utilization, future research will focus on the inclusion of additional topographical classifications of CP, additional gait phases and biomechanics data, and further characterization of gait deviations at cluster boundaries. Machine learning may provide important opportunities to investigate clinically important aspects of body structure and function and impairments including limb asymmetries, muscle spasticity, ageing as well as physical therapy and surgical intervention outcomes.
